# Metabolic Bone Disease in Captive Flying Foxes: A Conceptual Framework and Future Perspectives

**DOI:** 10.3390/metabo16010087

**Published:** 2026-01-21

**Authors:** Diana Faim, Isabel Pires, Filipe Silva

**Affiliations:** 1 Department of Veterinary Sciences, School of Agricultural and Veterinary Sciences (ECAV), University of Trás-os-Montes e Alto Douro, 5000-801 Vila Real, Portugal; dianasofaim@gmail.com (D.F.); fsilva@utad.pt (F.S.); 2 FozCanis—Hospital Veterinário da Figueira da Foz—Evidensia, 3080-230 Buarcos, Portugal; 3 Centre for Animal Sciences and Veterinary Studies (CECAV), Associate Laboratory of Animal and Veterinary Sciences—AL4AnimalS, University of Trás-os-Montes e Alto Douro, 5000-801 Vila Real, Portugal

**Keywords:** metabolic bone disease, *Pteropus*, vitamin D_3_, calcium–phosphorus homeostasis, UVB radiation, nutritional secondary hyperparathyroidism, captive management

## Abstract

In *Pteropus* spp., metabolic bone disease has been consistently associated with fruit-based diets that are deficient in calcium, vitamin D precursors, and protein, as well as limited ultraviolet B (UVB) exposure, as reported in zoological surveys and clinical observations. Comparative mammalian physiology suggests that dysregulation of the endocrine axis involving parathyroid hormone (PTH), fibroblast growth factor 23 (FGF23), calcitonin, and calcitriol may contribute to disease development, although direct species-specific endocrine data in flying foxes remain scarce. This narrative review synthesizes current knowledge from published zoological reports, clinical observations, and comparative mammalian physiology regarding the etiology, pathophysiology, and clinical expression of metabolic bone disease in captive flying foxes. Much of the available evidence is derived from juvenile *Pteropus vampyrus*, and its applicability to other *Pteropus* species remains to be fully established. The limited availability and consistency of existing data, together with the scarcity of controlled experimental and longitudinal studies, necessarily constrain the conclusions that can be drawn. Nevertheless, this review highlights key nutritional and environmental risk factors and summarizes evidence-informed preventive management strategies to improve skeletal health and welfare in managed *Pteropus* populations.

## 1. Introduction

Bats provide essential ecological functions that directly support human subsistence, yet they remain widely misunderstood. Although often perceived as nuisances or potential disease reservoirs, they contribute substantially to ecosystem stability. Depending on their feeding guild, bats support agricultural productivity and ecosystem functioning, suppress pest species, and reduce pesticide use, thereby enhancing economic output and public health [1,2].

Flying foxes (*Pteropus* spp.) are large frugivorous bats distributed across tropical and subtropical regions of Asia, Africa, and Oceania [3]. They play an essential ecological role as pollinators and seed dispersers, maintaining the structure and diversity of tropical forest ecosystems [1,4]. However, many *Pteropus* species are now threatened by habitat destruction, hunting, and climate change, leading to significant declines in wild populations [3,5,6,7]. Of the 65 recognized *Pteropus* species, six are already extinct, and the majority of the remainder are currently threatened, with 38 species classified as Vulnerable, Endangered, or Critically Endangered according to the IUCN Red List [8].

Zoological institutions have become critical refuges for conservation, rehabilitation, and ex situ research of threatened populations, namely *Pteropus* species [9]. However, maintaining tropical bat species in captivity, particularly in temperate climates, poses substantial physiological and husbandry challenges due to their specialized nutritional, physiological, and environmental needs. Diets must compensate for the limited nutritional value of cultivated fruits by including protein- and mineral-rich supplements. At the same time, water, vitamins, and key macro- and micronutrients must be provided in controlled amounts to avoid deficiencies or toxicities. Equally critical are enclosures designed to permit continuous flight, support natural behaviors, and provide thermal regulation, humidity control, ventilation, appropriate lighting, and sufficient resting sites with visual barriers. These requirements highlight the complexity of husbandry for *Pteropus* species and the importance of following evidence-based recommendations, such as those outlined by the Bat TAG Nutrition Subcommittee [10,11,12,13,14].

Over the past decade, multiple zoological facilities have reported that juvenile *Pteropus vampyrus*, the most commonly maintained Flying fox species in captivity, develop skeletal deformities and impaired flight during early growth [9]. These clinical observations are consistent with mineral and metabolic bone disorders, including nutritional or renal, chronic kidney disease–mineral bone disorder (CKD-MBD), with proposed etiologies including vitamin D deficiency and calcium–phosphorus imbalance [9,12,15,16].

This condition has attracted increasing attention from both researchers and zoological institutions because, when uncompensated, it leads not only to severe skeletal deformities and impaired locomotion but can ultimately fail to thrive and result in mortality, thereby compromising the long-term survival of captive individuals and, in threatened species, the success of ex situ conservation programs [9].

However, the true prevalence of this condition across zoological institutions, as well as its occurrence in other *Pteropus* species, remains unknown, and the underlying mechanisms are not yet fully characterized. To date, epidemiological data on metabolic bone disease in bats remain extremely limited, and the few detailed pathological and metabolic studies available have been conducted predominantly in microchiropteran bats rather than in megachiropteran bats such as flying foxes [9,17,18,19]. This reliance on microchiropteran data raises uncertainty as to whether metabolic bone disease in *Pteropus* spp. shares identical etiological mechanisms and whether vitamin D_3_ plays an equally central role in calcium absorption and mineral homeostasis in these species. Consequently, due to the absence of controlled dietary trials, UVB exposure experiments, and longitudinal biochemical studies in *Pteropus* species, current understanding of metabolic bone disease relies primarily on observational zoological reports, clinical case series, and extrapolation from comparative mammalian physiology.

Accordingly, this narrative review integrates evidence from comparative physiology, endocrinology, and zoo management to provide a conceptual framework for understanding metabolic bone disease in *Pteropus* spp. By synthesizing available data and explicitly acknowledging current limitations, this review aims to support evidence-informed husbandry strategies and identify key knowledge gaps relevant to the conservation and welfare of captive flying fox populations.

## 2. Metabolic Bone Disease in Flying Foxes

Metabolic bone disease is a systemic disorder characterized by defective bone mineralization resulting from imbalances in calcium, phosphorus, and vitamin D metabolism [20]. The condition encompasses several clinical entities, including rickets, osteomalacia, renal, and nutritional secondary hyperparathyroidism, all of which compromise skeletal integrity and function [16,21].

Although extensively described in reptiles and domestic mammals, the condition remains poorly characterized in chiropterans, particularly in frugivorous bats such as *Pteropus* spp. [9,15]. However, recent reports from zoological institutions have confirmed that captive flying foxes are highly susceptible to nutritional and metabolic bone disorders, particularly during early developmental stages, as described in other bat species [9].

Nutritional calcium–phosphorus imbalance, vitamin D deficiency, and inadequate UVB exposure have been implicated; however, species-specific pathways and the relative contributions of each factor remain poorly characterized. Most available knowledge derives from extrapolation from reptiles, domestic mammals, and human mineral disorders [22,23,24], while recent zoo-based reports merely confirm susceptibility without fully elucidating the cause [9]. Consequently, the mechanistic basis of MBD in flying foxes should be interpreted cautiously, acknowledging that its pathogenesis is likely multifactorial and not yet fully understood.

The term “Metabolic Bone Disease” is intentionally broad, reflecting both the spectrum of mineral and skeletal abnormalities observed and the current limitations in species-specific knowledge for chiropterans. Its use acknowledges that the condition arises from multiple interacting nutritional, endocrine, metabolic, and environmental factors rather than a single defined etiology. Accordingly, MBD in flying foxes should be approached through an integrated view of mineral metabolism, emphasizing the endocrine pathways that regulate calcium–phosphorus balance and vitamin D homeostasis, and highlighting the need for a multidimensional diagnostic approach when interpreting skeletal and metabolic abnormalities in captivity. This integrative perspective aligns with conceptual frameworks used in human nephrology, particularly Chronic Kidney Disease–Mineral and Bone Disorder (CKD-MBD), which is characterized by biochemical abnormalities (calcium, phosphorus, vitamin D metabolites, PTH, FGF23), skeletal lesions (renal osteodystrophy), and soft-tissue or vascular calcification [25,26]. Although renal dysfunction has not been confirmed as a primary cause of mineral imbalance in *Pteropus* spp., and vascular calcification has not been described in bats, the CKD-MBD framework remains a valuable comparative model for understanding the endocrine complexity underlying mineral disorders.

Despite the wide geographic distribution and ecological diversity of the genus *Pteropus*, most reports of metabolic bone disease originate from a limited number of species, particularly *P. vampyrus*, and from zoological institutions located in temperate regions. Consequently, reported husbandry challenges, such as dietary composition, UVB availability, enclosure design, and seasonal light exposure, may differ substantially from those encountered in tropical environments. Species-specific physiological differences, including growth rate, skin pigmentation, behavioral exposure to sunlight, and reproductive energetics, may further modulate susceptibility to mineral imbalance. These factors remain insufficiently studied and should be considered when extrapolating findings across species, regions, or climatic contexts [9,12,17,19].

### 2.1. Etiology of Metabolic Bone Disease

By understanding the mechanisms regulating calcium homeostasis and bone remodeling, it becomes possible to clarify the alterations underlying metabolic bone disease and to identify its most likely etiological drivers. Given the scarcity of species-specific experimental data in *Pteropus* spp., the proposed causes are primarily inferred from established principles of mammalian physiology and evidence from other taxa. Within this framework, metabolic bone disease is best interpreted as a multifactorial condition resulting from interactions among nutritional deficiencies, inadequate UVB exposure, physiological demands, organ dysfunction, and social dynamics within captive colonies. These etiological factors converge on the tightly regulated endocrine pathways that control calcium, phosphorus, and vitamin D metabolism, ultimately disrupting bone remodeling and leading to skeletal pathology [12].

One of the major contributing factors that should be considered in metabolic bone disease in captive flying foxes is prolonged dietary calcium deficiency, inadequate vitamin D_3_ intake, and/or imbalance of the calcium-to-phosphorus (Ca:P) ratio. In natural environments, bats of the genus *Pteropus* maintain a relatively balanced mineral intake by consuming diverse food sources, including fruits, flowers, pollen, and leaves, which supply both calcium and trace nutrients [10,27]. In contrast, captive diets depend heavily on cultivated fruits, which are carbohydrate-rich but deficient in calcium, protein, and vitamin D [28,29]. These fruits typically present an inverted Ca:P ratio (≈0.3:1), far below the physiological requirement of 1.5–2:1. Chronic ingestion of such diets predisposes animals to sustained hypocalcemia and relative hyperphosphatemia, triggering secondary nutritional hyperparathyroidism and accelerating bone resorption, thereby increasing the risk of skeletal deformities and impaired locomotion [16,30].

Ultraviolet B (UVB) deprivation represents a major etiological factor contributing to metabolic bone disease in captive *Pteropus* spp. Cutaneous synthesis of vitamin D_3_ depends on exposure to UVB wavelengths between 280–315 nm, which mediate the photochemical conversion of epidermal 7-dehydrocholesterol to previtamin D_3_, followed by temperature-dependent thermal isomerization to cholecalciferol. Effective vitamin D_3_ synthesis is therefore influenced by multiple environmental and biological modifiers, including latitude, season, time of day, solar zenith angle, atmospheric ozone concentration, skin pigmentation, and ambient temperature. Geographic location plays a critical role in UVB availability. Regions at low latitudes experience more consistent year-round UVB irradiance, whereas higher latitudes and temperate climates are characterized by marked seasonal reductions, particularly during winter months, when increased solar zenith angles result in greater atmospheric absorption of UVB radiation. Suboptimal ambient temperatures may further impair vitamin D_3_ synthesis by reducing the efficiency of thermal isomerization, even when UVB radiation is present. Consequently, institutions located outside tropical regions face greater challenges in maintaining adequate endogenous vitamin D_3_ production in captive flying foxes [19,31,32,33].

In captive settings, these limitations are frequently exacerbated by enclosure design. Natural sunlight exposure is often absent or significantly reduced by glass or acrylic barriers, which block more than 95% UVB radiation below 315 nm [31,32]. As a result, apparent exposure to daylight does not translate into effective cutaneous vitamin D_3_ synthesis. Artificial UVB-emitting lamps may partially compensate for this deficit; however, their efficacy is highly variable and depends on lamp spectrum, aging, positioning, distance, exposure duration, and animal behavior. Social structure within colonies further modulates UVB exposure. Dominant individuals preferentially occupy roosting sites with greater light exposure, leaving subordinate animals, often juveniles, with insufficient UVB [19]. Insufficient UVB exposure reduces endogenous vitamin D_3_ synthesis, thereby decreasing intestinal calcium absorption and promoting endocrine responses that directly contribute to MBD development [9,12,13,31,34].

Intra-colony variation in disease expression, with some individuals developing severe skeletal deformities while others remain subclinically affected, is further shaped by social hierarchy, as dominant individuals may monopolize food resources while subordinates experience reduced nutrient intake. Chronic social stress exacerbates skeletal pathology by elevating glucocorticoid levels, which suppress osteoblast activity and impair bone formation [9,12,13].

Developmental timing effects represent an additional and likely important modifier of susceptibility to MBD. Periods of increased physiological demand, particularly gestation, lactation, and rapid postnatal growth, amplify mineral requirements and exacerbate calcium imbalance when dietary intake or UVB exposure is inadequate. Experimental studies in microchiropteran bats have demonstrated intense bone resorption accompanied by loss of osteoid in *Myotis lucifugus* during periods of calcium stress, notably hibernation (when calcium intake ceases) and during pregnancy and lactation, when calcium demands are markedly increased [18]. Similar lactation-associated reductions in trabecular bone area within long-bone epiphyses have been reported, highlighting the vulnerability of the maternal skeleton during reproductive investment [35].

Accelerated juvenile growth further increases susceptibility to MBD under suboptimal nutritional or environmental conditions. The disease has been documented in prematurely weaned and hand-reared bats, where inadequate calcium supply from maternal milk or nutritionally inappropriate milk replacers has been implicated, even when supplementation is attempted. These observations suggest that early-life mineral deficiency may result in irreversible impairment of skeletal development [15,36].

Organ dysfunction may further modify disease expression. Both hepatic and renal tissues are essential for the hydroxylation steps that convert vitamin D_3_ into its biologically active form, calcitriol (1,25-dihydroxyvitamin D_3_) [12]. In chronic renal dysfunction, phosphate retention and reduced calcitriol synthesis lead to persistent hypocalcemia and continuous stimulation of parathyroid hormone (PTH) secretion, establishing a self-perpetuating cycle of bone resorption and mineral depletion [24,37,38], as described in CKD-MBD [24,25,26].

Alternative etiological mechanisms described in other mammalian taxa merit consideration. Genetic disorders affecting vitamin D metabolism, including defects in renal 1α-hydroxylase activity or resistance at the vitamin D receptor level, are well-recognized causes of rickets in humans [39,40] and domestic animals [41,42,43]. Similarly, inherited hypophosphatemic disorders associated with dysregulated FGF23 signaling and renal phosphate wasting have been described [44,45,46]. To date, however, no genetic or inherited metabolic bone disorders have been documented in *Pteropus* spp., and their relevance remains speculative. Nonetheless, these mechanisms highlight the potential for inter-individual or interspecific variability in mineral and endocrine regulation and reinforce the need to consider non-nutritional contributors when interpreting disease expression in captive populations.

### 2.2. Pathophysiological Basis of Metabolic Bone Disease

As described in etiology, current understanding of the pathophysiology of metabolic bone disease in flying foxes is incomplete, and proposed mechanisms are primarily inferred from other mammalian taxa. Direct measurements of endocrine regulators such as parathyroid hormone (PTH), fibroblast growth factor 23 (FGF23), and calcitonin in *Pteropus* species are scarce, limiting species-specific interpretation.

Bone tissue is metabolically active and continuously remodeled by the coordinated activity of osteoblasts, osteoclasts, and osteocytes [30]. Osteoblasts synthesize the organic matrix (osteoid), which is later mineralized by calcium and phosphate deposition to form hydroxyapatite crystals. Osteoclasts resorb mineralized bone, releasing calcium and phosphate into the extracellular fluid, while osteocytes act as mechanosensors and regulators of bone turnover via paracrine signaling. These processes depend on tightly regulated mineral homeostasis, mediated by the integrated function of bone, kidneys, and intestine [12,47,48].

Calcium and phosphorus homeostasis is primarily regulated by the coordinated actions of PTH, calcitriol (1,25-dihydroxyvitamin D_3_), and FGF23. PTH mobilizes calcium and phosphorus from bone, enhances renal calcium reabsorption, and increases phosphate excretion. Calcitriol, in turn, increases intestinal absorption of both minerals and modulates gene expression in osteoblasts and osteoclasts, thereby influencing bone remodeling dynamics [49]. Any disturbance in this network, whether caused by dietary mineral imbalance, UVB deprivation that impairs cutaneous vitamin D synthesis, or renal dysfunction that limits phosphate excretion, can destabilize the entire system and drive the development of mineral and metabolic bone disease in bats [49,50].

Calcium availability represents an essential determinant of skeletal integrity. Within the body, calcium exists in three principal forms: ionized (free), protein-bound (primarily to albumin), and complexed with anions such as phosphate, forming hydroxyapatite crystals within the bone matrix. The ionized fraction represents the biologically active form and is the primary determinant of calcium homeostasis, as it is independent of protein binding and directly sensed by regulatory mechanisms. Approximately 99% of total body calcium is stored in bone, while smaller fractions are distributed within soft tissues and the extracellular fluid. Despite its relatively low volume, the extracellular calcium pool is critical for physiological function and tight regulation of serum calcium concentrations [51,52,53].

Intestinal absorption of dietary calcium accounts for approximately 20% of intake and occurs via both passive diffusion and active, vitamin D_3_–dependent transport mechanisms. Active transport becomes increasingly important when dietary calcium availability is low and when adequate levels of calcitriol (1,25-dihydroxyvitamin D_3_) are required [54,55]. Calcium absorption efficiency is further influenced by dietary composition and gastrointestinal conditions. Adequate vitamin D_3_ status, acidic luminal pH, and low circulating calcium concentrations enhance absorption, whereas oxalates, phytates, excessive insoluble fiber, and high dietary phosphorus or magnesium relative to calcium reduce calcium bioavailability by competing for vitamin D-dependent transport mechanisms [12,56,57].

Renal handling of calcium further contributes to its systemic regulation. Approximately 98% of filtered calcium is reabsorbed along the proximal and distal renal tubules, while bone serves as the primary site of calcium exchange through continuous remodeling. During the final stage of bone formation, calcium is incorporated into hydroxyapatite crystals to mineralize the organic osteoid matrix. Inadequate calcium availability disrupts this process, leading to defective mineralization and the development of rickets or osteomalacia. Maintenance of stable serum calcium concentrations is therefore critical for skeletal integrity [54].

Calcium homeostasis is regulated by the coordinated actions of parathyroid hormone (PTH), calcitonin, and calcitriol (1,25-dihydroxyvitamin D_3_). When hypocalcemia develops, reduced activation of the calcium-sensing receptor (CaSR) on parathyroid cells triggers sustained PTH secretion. PTH increases bone resorption by stimulating osteoclast activity, enhances renal calcium retention, and promotes phosphate excretion. It also upregulates renal 1α-hydroxylase, increasing calcitriol production and thereby improving intestinal absorption of both minerals [38,51,58]. With persistent stimulation, the parathyroid glands may undergo hyperplasia, leading to abnormal and autonomous PTH secretion. This enlargement is accompanied by reduced expression of CaSR and vitamin D receptors, reinforcing the cycle of secondary hyperparathyroidism. Chronically elevated PTH accelerates bone turnover and releases additional calcium and phosphorus into circulation, further destabilizing mineral homeostasis. Although continuously high PTH levels drive bone loss, intermittent PTH signaling can support osteoblast differentiation, illustrating its dual role in bone remodeling [59,60].

In contrast, hypercalcemia suppresses PTH secretion, increases renal calcium excretion, and limits further intestinal calcium absorption, thereby favoring skeletal mineral retention. Magnesium also modulates calcium homeostasis by affecting PTH secretion and action. Mild hypomagnesemia stimulates PTH release, whereas severe magnesium deficiency paradoxically suppresses PTH secretion and induces skeletal resistance to PTH, potentially resulting in hypocalcemia and impaired bone mineralization [38,58,61,62].

Phosphorus metabolism is closely interconnected with calcium regulation [38,51]. As with calcium, under normal physiological conditions, phosphorus balance is maintained through coordinated regulation of intestinal absorption, renal excretion, and exchange with bone stores. Most dietary phosphorus is absorbed in the small intestine, while the kidneys fine-tune phosphate elimination in response to endocrine signals, primarily PTH and FGF23. Both hormones promote phosphaturia and downregulate calcitriol activation, thereby limiting intestinal phosphate uptake and stabilizing the systemic phosphate pool [49]. Additionally, klotho functions as an essential co-receptor that enables FGF23 signaling in the kidney. By forming a complex with specific FGF receptors, predominantly FGFR1, it increases their affinity for circulating FGF23, allowing the hormone to exert its phosphaturic effects. Although FGF23 can also interact with FGFR2 and FGFR3, these interactions are weaker and contribute less to phosphate regulation. When FGF23 concentrations become markedly elevated, the hormone may bind to FGFR4 in a klotho-independent manner, a pathway associated with off-target systemic effects rather than mineral homeostasis [49,63,64].

When phosphorus levels rise, FGF23 is rapidly upregulated by osteocytes and acts as a central regulator of phosphate metabolism. In addition to its phosphaturic effects, FGF23 strongly suppresses renal 1α-hydroxylase activity, reducing calcitriol synthesis and, consequently, decreasing intestinal absorption of calcium and phosphorus. Although this response is protective against hyperphosphatemia, chronically elevated FGF23 induces a persistent low-calcitriol state that exacerbates hypocalcemia and maintains secondary hyperparathyroidism. This hormonal profile increases osteoclast activity, accelerates bone resorption, and contributes directly to the osteopenic and osteomalacic lesions characteristic of MBD. As phosphorus levels continue to rise, FGF23 secretion intensifies to prevent hyperphosphatemia and to inhibit calcitriol synthesis. The resulting hypocalcemia perpetuates PTH stimulation, creating a self-reinforcing cycle of bone demineralization [49,65,66]. Persistent activation of this pathway may lead to osteitis fibrosa, also called fibrous osteodystrophy, characterized by replacement of mineralized bone with fibrocollagenous tissue, reduced mechanical strength, and skeletal deformity [16,21].

Vitamin D_3_ metabolism also could play a central role in the pathogenesis of MBD. In mammals, 7-dehydrocholesterol in the epidermis is photoconverted to previtamin D_3_ under UVB radiation (280–315 nm), which then thermally isomerizes to cholecalciferol [34]. This precursor undergoes two hydroxylations: first in the liver by 25-hydroxylase (CYP2R1) to produce 25(OH)D_3_ (calcidiol), and then in the kidney by 1α-hydroxylase (CYP27B1) to yield 1,25(OH)_2_D_3_ (calcitriol), the biologically active form. Calcitriol enhances calcium and phosphorus absorption in the intestine and regulates bone remodeling by acting on osteoblast and osteoclast gene expression. The catabolic enzyme CYP24A1 degrades both 25(OH)D_3_ and 1,25(OH)_2_D_3_, serving as a key safeguard against vitamin D toxicity; this enzyme has been sequenced in several mammalian species, including bats, and mutations in humans are associated with hypercalcemia and renal disease [34,54,67].

Experimental studies in frugivorous bats demonstrate that these species retain functional photochemical pathways for vitamin D_3_ synthesis when UVB is available. In *Pteropus* spp., however, baseline plasma concentrations of 25(OH)D_3_ are consistently low (≈1.5 ng/mL), and 1,25(OH)_2_D_3_ levels fall at the lower end of the mammalian range. Sunlight-exposure trials in these bats further show that individuals can increase circulating 25(OH)D following controlled solar exposure, confirming that endogenous synthesis is possible when UVB reaches the skin. However, ecological habits and indoor housing conditions restrict activation of this pathway almost entirely in captivity, predisposing *Pteropus* colonies to hypovitaminosis D and downstream disturbances of calcium–phosphorus homeostasis [11,19]. Many megachiropteran species roost in dark environments, and captive bats are frequently housed indoors behind glass or acrylic barriers that block nearly all UVB wavelengths (<315 nm), preventing cutaneous photoconversion of 7-dehydrocholesterol. Although artificial UVB lamps may partially compensate, their effectiveness depends on lamp aging, UVB output, positioning, and behavioral factors that influence access to irradiated areas [31].

Pigmentation is another factor influencing vitamin D cutaneous synthesis. Melanin effectively absorbs UVB radiation, decreasing the amount available for vitamin D_3_ photoproduction [68]. Therefore, dark-skinned fruit bats require longer exposure times to synthesize adequate vitamin D_3_ levels compared with lighter individuals [19].

In addition to cutaneous synthesis, dietary intake may contribute to vitamin D status in bats, although this pathway remains poorly characterized. Frugivorous diets are generally low in vitamin D; however, incidental ingestion of insects, pollen, or vitamin D–containing plant components, as well as maternal transfer during gestation or lactation, may represent supplementary sources [10]. The relative contributions of these alternative pathways to overall vitamin D homeostasis in *Pteropus* species remain unknown and warrant further investigation.

Vitamin D_3_ deficiency reduces calcium absorption, leading to persistent hypocalcemia and compensatory hyperparathyroidism [30]. The prolonged elevation of PTH accelerates bone resorption, resulting in structural weakening and fibrous tissue infiltration, hallmark features of MBD [21]. In juveniles, this impaired mineralization manifests as rickets, characterized by widened and irregular growth plates, metaphyseal swelling, and bone deformity [16]. In adults, the same defect leads to osteomalacia, with accumulation of unmineralized osteoid and increased fracture susceptibility [30].

In chronic kidney disease, reduced glomerular filtration impairs phosphate excretion, resulting in progressive phosphate retention. Sustained hyperphosphatemia stimulates increased secretion of fibroblast growth factor 23 (FGF23), which initially acts as a compensatory mechanism by promoting phosphaturia but ultimately suppresses renal 1α-hydroxylase activity, leading to reduced calcitriol synthesis and diminished intestinal absorption of calcium and phosphorus [37,69,70,71]. The resulting hypocalcemia further stimulates PTH secretion, leading to continuous bone reabsorption. Hypocalcemia is exacerbated by hyperphosphatemia through the inverse relationship between calcium and phosphorus and the low solubility of ionized phosphate in the bloodstream. Elevated phosphate binds ionized calcium, forming calcium–phosphate complexes and reducing the circulating biologically active calcium fraction. This decline in serum calcium perpetuates excessive PTH secretion, leading to sustained bone resorption and the release of calcium and phosphate from the skeletal matrix. Progressive skeletal calcium depletion leads to reduced bone mineral density, increased fragility, skeletal deformities, and pathological fractures. Over time, mineralized bone is replaced by fibrocollagenous tissue, a hallmark of fibrous osteodystrophy [16,21,65].

In advanced renal impairment, phosphate excretion becomes inadequate despite markedly elevated FGF23 and PTH. This transition from adaptive to maladaptive endocrine control results in progressive phosphorus retention, sustained calcitriol suppression, and chronic hypocalcemia, driving PTH hypersecretion [49]. Also, klotho levels decline, and the kidney becomes less responsive to FGF23, reducing its phosphaturic efficiency. This diminished sensitivity promotes phosphate retention and further disturbs mineral balance. Beyond its role in FGF23 signaling, klotho also influences cellular stress responses, and its deficiency has been associated with broader systemic dysfunction [64,72,73].

**Figure 1:**
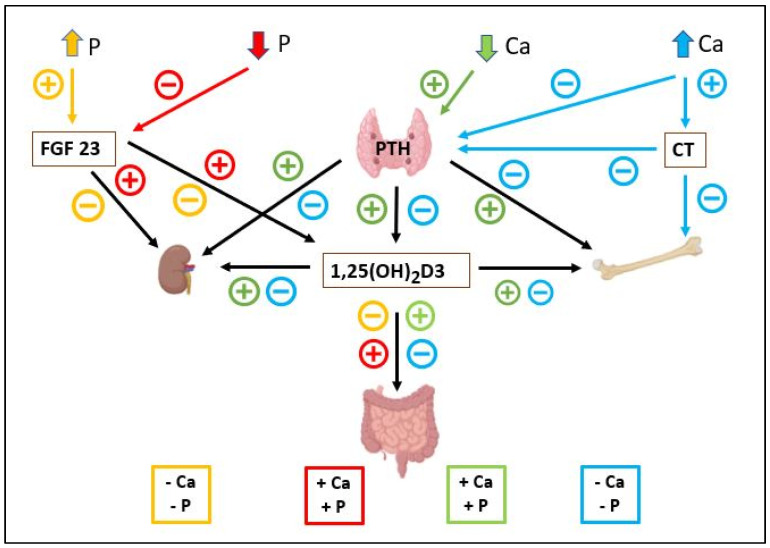
Integrated endocrine pathways controlling Ca–P balance. Fibroblast growth factor 23 (FGF23), PTH, calcitonin (CT), and 1,25(OH)_2_D_3_ coordinate renal, intestinal, and skeletal responses to maintain calcium (Ca) and phosphorus (P) levels. Colors represent the effects of initial phosphorus level increase (yellow) or decrease (red), and calcium level increase (blue) or decrease (green).Arrows represent stimulatory (+) or inhibitory (-) effects.

Figure 1 resumes the interdependence of calcium and phosphorus metabolism. Both minerals share overlapping regulatory mechanisms mediated by PTH, FGF23, and calcitriol [65]. High dietary phosphorus not only suppresses calcium absorption but also stimulates FGF23 secretion, thereby further inhibiting vitamin D activation and perpetuating hypocalcemia [74]. Conversely, calcium deficiency amplifies PTH release, accelerating bone resorption and phosphorus excretion [38]. This reciprocal relationship illustrates the delicate balance required for skeletal homeostasis, a balance easily disrupted in bats by nutritional inadequacy, inappropriate husbandry, or environmental deprivation [12,13,34].

### 2.3. Clinical Signs

Clinical manifestations of Metabolic Bone Disease (MBD) primarily reflect the progressive loss of bone mineralization and the resulting structural fragility. Although the underlying metabolic imbalance is systemic, the most evident effects are musculoskeletal, with visible deformities and functional impairment. The disease typically affects juveniles during growth, when calcium and phosphorus demands are highest, but adults maintained under poor nutritional or environmental conditions may also develop milder or chronic lesions. The onset is often insidious, with subtle behavioral changes preceding overt skeletal abnormalities [9,12,15].

The earliest signs are behavioral and functional, including reduced flight activity, clumsy landings, reluctance to hang, and prolonged resting periods. These manifestations are probably due to generalized weakness, pain, or impaired bone integrity. As the disease progresses, affected bats develop skeletal deformities of the appendicular and craniofacial skeleton. Bowing of the radius, ulna, and humerus is frequently observed, accompanied by joint thickening and palpable swelling along the long bones. Mandibular and maxillary deformities are common, leading to malocclusion, difficulty chewing, and secondary weight loss due to reduced food intake. The so-called “rubber jaw” appearance, described in other mammals with osteitis fibrosa, has also been documented in *P. vampyrus*, associated with cortical thinning and fibrous replacement of alveolar bone [9,12,21].

**Figure 2:**
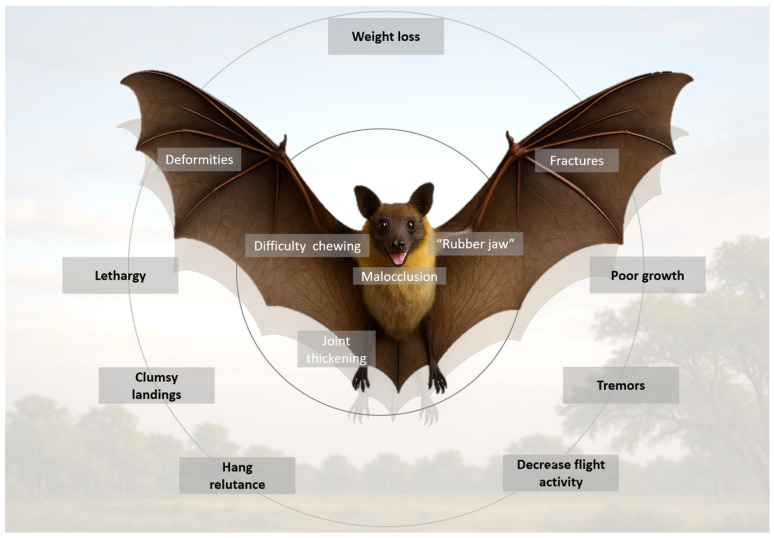
Main clinical signs of metabolic bone disease in Flying foxes.

Neuromuscular signs may develop in advanced stages. Hypocalcemia disrupts neuromuscular excitability, leading to tremors, spasms, and tetanic contractions. In severe cases, vertebral deformities or pathological fractures can compress the spinal cord, producing paresis or paralysis. Chronic pain due to skeletal deformities and fractures contributes to lethargy, anorexia, and progressive weight loss, creating a self-reinforcing cycle of malnutrition and mineral depletion [12,15,21]. Figure 2 summarizes the main clinical signs of metabolic bone disease in Flying foxes.

Except for the recent multi-institutional questionnaire survey by Faim et al. [9], all studies reporting on metabolic bone disease and calcium regulation in bats have been conducted exclusively in *Microchiropteran* species. Metabolic bone disease has been identified in prematurely weaned or hand-reared insectivorous bats, including *Tadarida brasiliensis*, *Lasiurus borealis*, and *Nycticeius humeralis*, typically following maternal rejection. The condition is painful, and affected pups may vocalize when handled [36]. Additional cases have been reported involving two *Desmodus rotundus* juvenile bats with rickets of undetermined etiology [75]. This taxonomic limitation introduces uncertainty regarding the direct applicability of these findings to megachiropterans, particularly flying foxes (*Pteropus* spp.). Extrapolation of these clinical signs to *Pteropus* spp. should therefore be undertaken with caution, while acknowledging that they currently represent the best available scientific basis for interpreting the clinical signs and skeletal alterations observed in captive flying foxes.

### 2.4. Diagnosis

Diagnostic approaches to mineral and bone disorders vary widely across species and are strongly influenced by the availability and validation of analytical tools. In mammals, evaluation typically relies on a combination of biochemical markers (calcium, phosphate, 25(OH)D, parathyroid hormone, and bone turnover markers) and skeletal imaging. These methods allow detection of disturbances in mineral metabolism and structural bone abnormalities, but remain limited in sensitivity for early or low-grade lesions [24].

In human medicine, the Kidney Disease: Improving Global Outcomes (KDIGO) framework emphasizes a multidimensional diagnostic strategy that integrates biochemical assessment; skeletal imaging using radiographs and dual-energy X-ray absorptiometry (DEXA), bone histomorphometry to characterize turnover, mineralization, and microarchitecture; and cardiovascular imaging to detect vascular and valvular calcification. This approach underscores the systemic nature of CKD-MBD and highlights that isolated biochemical or radiographic findings provide an incomplete diagnostic picture [25,76]. A similar multidimensional structure could, in principle, be adapted to non-human species, including bats, as analytical tools and species-specific reference intervals become available.

Radiographic examination currently provides the main diagnostic support. However, in the early stages of disease, radiographs often appear normal, as 30–50% mineral loss is required before changes become detectable [16]. With disease progression, generalized osteopenia, cortical thinning, loss of trabecular density, metaphyseal irregularities, poorly defined growth plates, and mandibular demineralization may develop. In severe cases, pathological fractures and pseudofractures may develop along stress-bearing regions such as the humerus, radius, or femur. Cranial radiographs reveal decreased mandibular density and resorption of alveolar margins [15,16]. Computed tomography and DEXA provide more sensitive and quantitative assessments of bone mineral content, although baseline values for bats remain undefined [77].

Histopathology, therefore, could help confirm the diagnosis and assess the severity of mineral and bone disorders across species. Osteitis fibrosa helps identify secondary hyperparathyroidism and CKD-Metabolic Bone Disease. It is characterized by fibro-osteoclastic tunneling resorption resulting from chronically elevated PTH levels [70]. The cortical bone becomes thin and porous, while marrow spaces expand due to fibrous proliferation. In juvenile bats, the growth plates show disorganized chondrocyte columns, irregular endochondral ossification, and metaphyseal swelling typical of rickets. Osteoclastic resorption is pronounced along trabecular and cortical surfaces, reflecting the hyperparathyroid state, whereas osteoblasts often appear flattened or inactive, indicating reduced bone formation [16,78]. In *Desmodus rotundus*, two juvenile bats diagnosed with rickets exhibited abnormal growth plates associated with a reduced number of osteoblasts, absence of osteoid, and decreased metaphyseal trabeculae [75]. However, histopathological alterations in metabolic bone disease generally reflect chronic or advanced pathology and may not capture early or subclinical disturbances in mineral metabolism. As recognized in systemic disorders such as chronic kidney disease–mineral and bone disorder, structural bone changes, biochemical abnormalities, and extra-skeletal manifestations (including vascular or soft tissue mineralization) may evolve asynchronously and do not necessarily correlate linearly [26,76]. Consequently, isolated histopathological findings may underestimate the systemic nature of mineral and bone disorders. Moreover, overlapping histologic features, such as osteitis fibrosa, may be observed in distinct pathological contexts, including nutritional secondary hyperparathyroidism and CKD-MBD, thereby limiting diagnostic specificity when histology is interpreted in isolation. Accordingly, histopathology should be regarded as a supportive component within a multimodal diagnostic framework integrating clinical assessment, biochemical profiling, imaging, and husbandry context, rather than as a definitive indicator of disease etiology [26].

Biochemical assessment supports the diagnosis and helps distinguish between nutritional and metabolic causes. Affected bats generally exhibit low serum calcium, elevated inorganic phosphorus, and high alkaline phosphatase (ALP) activity, indicating increased osteoblastic turnover and defective mineralization. Serum 25-hydroxyvitamin D_3_ (calcidiol) concentrations are typically low, confirming vitamin D deficiency, while parathyroid hormone (PTH) levels are elevated, consistent with secondary nutritional hyperparathyroidism [12,21]. In *Rousettus aegyptiacus*, available data suggest baseline values of approximately 4–6 ng/mL for 25(OH)D_3_, ~9 mg/dL for total serum calcium, and substantially lower PTH concentrations in individuals with adequate UVB exposure (~60 pg/mL), compared to markedly elevated values (>150 pg/mL) in vitamin-D-deficient nocturnal animals. These ranges, although not formal reference intervals, provide clinically useful comparative benchmarks for interpreting mineral metabolism in bats [79].

Evaluation of hepatic and renal function is essential to exclude secondary causes such as CKD-MBD and other organ dysfunction that could impair vitamin D hydroxylation. Persistent hypocalcemia despite adequate supplementation may indicate concurrent renal phosphate retention and calcitriol synthesis suppression [12,21].

Despite these tools, which are mainly adapted from other species, diagnosis remains challenging. The lack of validated assays, reference intervals, and feasible advanced imaging modalities constrains diagnostic accuracy. Radiographs have poor sensitivity for early bone changes; assays for PTH, FGF23, and bone turnover markers are often unavailable; and bone biopsies are difficult in small wildlife taxa. Consequently, many methods provide only indirect or low-resolution insights into bone metabolism, complicating precise classification.

These gaps highlight the need for standardized reference values, species-specific biomarker validation, and the development of imaging approaches adapted to small-bodied animals. Incorporating principles from the KDIGO diagnostic structure [26,76] may also support a more comprehensive evaluation of mineral metabolism and bone health across taxa.

Comparative data from other mammals, reptiles, and chiropterans often require extrapolation, introducing uncertainty into interpretation. Furthermore, similar clinical and biochemical profiles may arise from different etiologies, including nutritional deficiency, CKD-Metabolic Bone Disease, and endocrine dysfunction, thereby reinforcing the need for an integrative diagnosis that combines clinical, imaging, biochemical, and histopathological evidence. Standardized diagnostic protocols in zoological collections are essential, as misclassification can lead to inappropriate treatment and recurrence [9].

Environmental and nutritional evaluation forms an integral part of diagnosis. A detailed assessment of diet composition allows identification of imbalanced calcium–phosphorus ratios or a lack of vitamin D supplementation. Inspection of enclosures ensures that animals receive adequate UVB exposure, either through direct sunlight or through properly installed artificial lighting emitting within the 280–315 nm spectrum. Behavioral observations can reveal social dominance effects on feeding and light access [12,15].

### 2.5. Differential Diagnosis

Differential diagnosis of Metabolic Bone Disease in flying foxes requires careful distinction from other conditions that alter skeletal structure, mineral density, or bone metabolism. Because MBD is a systemic and progressive disorder, its clinical presentation often overlaps with a variety of nutritional, endocrine, infectious, neoplastic, and toxic bone pathologies. Accurate differentiation depends on integrating clinical, radiographic, biochemical, and histopathological findings with environmental and dietary assessments [9].

Vitamin C deficiency (scurvy), which can be pretty common in bats if they do not have an adequate diet, can mimic early MBD through impaired collagen synthesis and capillary fragility, leading to joint swelling, bone pain, and hemorrhages. Histologically, scurvy is characterized by defective osteoid formation with subperiosteal hemorrhage, while calcium and phosphorus metabolism remain normal. Differentiation, therefore, depends on biochemical normality of mineral levels and the presence of hemorrhagic lesions rather than true demineralization [80,81].

Copper and manganese deficiency are other differential diagnoses. This deficiency tends to cause similar signs, given their role in collagen synthesis [16,82].

Deficiencies are not the only cause of bone disorders. Toxicity from excess amounts of specific vitamins, such as A and D, results in narrow cortices and enlargement of the metaphyseal region in long bones, mimicking MBD. The way to distinguish between them is to perform laboratory tests for vitamins, calcium, and phosphorus, which, in hypervitaminosis D, will be elevated, unlike in MBD [16,78,83].

Chronic exposure to heavy metals, such as lead, thallium, and barium, can interfere with bone metabolism and mineralization. Lead poisoning inhibits osteoblastic activity and disrupts vitamin D metabolism, but radiographically produces metaphyseal “lead lines,” a feature absent in MBD [16,84,85,86]. Fluorosis, for instance, induces abnormal periosteal bone formation and cortical thickening, resulting in dense, brittle bones, in contrast to the demineralization pattern of MBD [16,87].

Hypertrophic osteodystrophy is a bone disorder exclusively affecting growing animals, whose extremities closely resemble those of MBD. They present pronounced, hot, painful, and rigid edema of both distal extremities of the long bones, symmetrically. The main radiographic feature is the presence of a radiolucent line parallel to the epiphyseal plate, and bone density is usually highly irregular, revealing alternating areas of radiopacity and radiolucency, with a visible thickening of the metaphyseal region [16,78,88,89].

Other differential diagnoses include polyostotic osteochondroma, a benign bone tumor of genetic origin, characterized by excessive hyaline cartilage formation around the growth plates. It manifests in young animals, with the most common lesions being bilateral, symmetrical metaphyseal lesions in long bones. Exostosis (excessive bone growth) progresses during growth, resulting in metaphyseal enlargement that becomes permanent after cartilage ossification [16,90,91].

### 2.6. Treatment

The management of Metabolic Bone Disease in captive flying foxes aims to restore mineral balance, alleviate clinical signs, and address environmental and nutritional deficiencies that precipitate the condition. Because MBD develops gradually due to chronic disturbances in calcium, phosphorus, and vitamin D metabolism, successful treatment requires an integrated approach combining immediate clinical stabilization with long-term preventive husbandry reforms. In most cases, recovery is partial, as skeletal deformities that occur during growth are irreversible; therefore, prevention remains the most effective strategy [12].

Therapeutic intervention begins with nutritional correction, targeting restoration of an optimal calcium-to-phosphorus (Ca:P) ratio and reestablishment of adequate vitamin D levels. Dietary analysis should be conducted to identify imbalances or deficiencies. Cultivated fruits such as banana, apple, and melon, though energy-rich, contain very low calcium and high phosphorus concentrations, resulting in an inverted Ca:P ratio. This imbalance must be corrected by incorporating calcium-rich foods such as leafy greens, figs, flowers, and pollen, as well as protein-enriched supplements formulated for frugivorous mammals. Commercial primate or omnivore pellets are particularly valuable, as they provide balanced mineral and vitamin D_3_ content when offered in small proportions alongside natural fruit [10,12].

Calcium supplementation may be achieved using calcium carbonate or calcium gluconate, administered orally or parenterally, depending on clinical severity. In acute cases presenting with tremors, paralysis, or seizures, intramuscular or subcutaneous injections of diluted calcium gluconate (10%) can rapidly correct hypocalcemia, with fluid therapy to maintain electrolyte stability. Once the animal stabilizes, long-term supplementation should continue orally, combined with dietary reform to ensure sustainable mineral intake [15].

Vitamin D supplementation is essential, particularly for colonies housed indoors without direct sunlight [31]. Although vitamin D can be provided as either D_2_ (ergocalciferol) or D_3_ (cholecalciferol), species differ markedly in their ability to use these forms. Old World primates can metabolize both [92], whereas New World primates and birds exhibit poor physiological utilization of D_2_, supporting the routine use of cholecalciferol (vitamin D_3_) in supplemented diets [93,94,95,96]. In bats, there is no evidence of an effective physiological use of vitamin D_2_, and current husbandry and veterinary protocols consistently rely on cholecalciferol (vitamin D_3_) [17]. Cholecalciferol (vitamin D_3_) can be administered orally or via fortified feed under veterinary supervision to avoid hypervitaminosis D. Because dietary sources alone may not restore physiological levels, environmental supplementation with ultraviolet B (UVB) exposure is critical. Natural sunlight remains the most effective and biologically appropriate UVB source, providing the spectral range (280–315 nm) necessary for cutaneous photoconversion of 7-dehydrocholesterol to previtamin D_3_. Where outdoor access is impractical, artificial UVB-emitting lamps designed for reptile or bird enclosures can be installed to replicate solar radiation [31].

Enclosures should provide sufficient vertical and horizontal space to allow free flight, essential for maintaining mechanical bone stimulation and preventing disuse osteopenia [97]. Pain management and orthopedic stabilization are often necessary to improve comfort and mobility. Analgesics should be administered as part of supportive care [15], while fractures require surgical or splint stabilization, depending on severity and location [12].

Pharmacological therapy targeting parathyroid hormone (PTH) suppression may be considered in advanced cases of secondary hyperparathyroidism. In human and veterinary medicine, vitamin D receptor activators (VDRAs) such as calcitriol or paricalcitol, and calcimimetics such as cinacalcet hydrochloride, have been used to reduce PTH secretion. However, these compounds carry risks of hypercalcemia and are not yet validated for use in chiropterans. Future studies are required to evaluate their safety and efficacy in bats [69].

### 2.7. Preventive Management Strategies

In addition to treatment, prevention is the cornerstone of long-term management and requires integrating nutritional science with environmental engineering. At present, no validated species-specific thresholds for ultraviolet B (UVB) irradiance, dietary calcium-to-phosphorus ratios, or endocrine biomarkers predictive of the onset of metabolic bone disease have been established for *Pteropus* species. Available quantitative recommendations are therefore derived primarily from zoological husbandry guidelines and from extrapolations from other mammals, rather than from experimental validation in flying foxes [10,98]. Similarly, although UVB wavelengths between 280–315 nm are required for cutaneous vitamin D synthesis, critical irradiance levels, exposure durations, and distance-dependent thresholds have not been defined for *Pteropus* spp. [10]. Consequently, preventive strategies should focus on optimizing diet, environment, and social structure to minimize risk factors.

Nutritional management represents one of the most critical components of disease prevention. Based on existing husbandry guidelines, dietary calcium levels of approximately 0.5–1% dry matter, phosphorus levels of 0.4–0.9% dry matter, and a Ca:P ratio close to 1.5–2:1 are commonly proposed as preventive targets in captive *Pteropus* management, with vitamin D supplementation (approximately 0.2–2 IU/g diet) recommended when UVB exposure is limited [99]. However, given current knowledge of minimum or recommended levels of key nutrients and minerals, it is prudent to formulate diets that meet these requirements and, when necessary, to supplement the diet to prevent deficiencies [10]. Diets should be diversified to include natural plant components such as figs, leaves, flowers, and pollen, thereby more closely reflecting the wild nutritional ecology. However, cultivated fruits alone are nutritionally insufficient, particularly with respect to calcium, protein, and several micronutrients, and therefore require supplementation to prevent metabolic bone disease. Successful captive feeding regimes incorporate complementary items such as leafy greens, vegetables, flowers, pollen, fruit nectar, and commercial pelleted diets formulated for primates or frugivorous birds, which provide higher protein levels, balanced calcium and phosphorus content, and essential vitamins, including vitamin D_3_ [10,13,100]. Although flying foxes are primarily frugivorous, wild individuals have been shown to supplement their diet with insects and ectoparasites, providing additional protein and trace minerals, particularly during periods of fruit scarcity [101,102,103]. Nevertheless, insects are poor sources of calcium and exhibit low Ca:P ratios, reinforcing the need for calcium-rich plant matter or targeted supplementation in captivity [104,105].

The method of food presentation also influences nutrient intake and disease risk. Chopping and mixing food items reduces selective feeding and reduces the risk of mineral imbalances. Feeding enrichment strategies, including puzzle feeders, suspended food items, and varied presentation formats, promote natural foraging behavior, physical activity, and skeletal loading, indirectly supporting bone health [10,13].

Periodic laboratory analysis of food composition is recommended to verify mineral and vitamin content. Feeding enrichment strategies, including suspended fruit items or scatter feeding, encourage natural foraging behavior, reduce stress, and promote muscular activity, all of which support skeletal health [13,97].

Light management and UVB exposure are equally critical for prevention. Daily lighting regimes should approximate natural photoperiods, typically 12 h of light and 12 h of darkness, to support circadian regulation [81]. Outdoor enclosures are preferable whenever feasible, as they provide access to natural sunlight. When indoor housing is unavoidable, access to natural daylight through windows or translucent panels should be maximized, although these do not substitute for UVB exposure. Artificial lighting systems should be regulated using timers and dimmers to simulate natural dawn–dusk transitions [10,13]. UVB-emitting lamps designed for birds or reptiles should be used where natural sunlight is insufficient; these systems require regular replacement and monitoring to ensure sustained UVB output. Periodic evaluation of enclosure layout and light-exposure zones is recommended to minimize shaded areas where UVB availability may be inadequate to support effective vitamin D synthesis [13,97].

Regarding life stage, juveniles, pregnant females, and lactating bats require higher mineral intake and should be regarded as high-risk groups for MBD. Juvenile flying foxes are particularly vulnerable due to rapid skeletal growth, as accelerated growth rates may disproportionately increase mineral requirements and amplify susceptibility to disease under suboptimal dietary or environmental conditions. However, growth trajectories may vary substantially among individuals depending on factors such as birth timing, maternal nutritional status, and postnatal feeding success. Reproductive status should likewise be considered a sensitive physiological period rather than a uniform risk category. Mineral requirements vary across gestation and lactation and may be further influenced by litter size, lactation duration, and interbirth interval. Prolonged or energetically demanding lactation periods may exacerbate maternal mineral depletion, particularly when dietary calcium availability, vitamin D status, or UVB exposure are limited. Although species-specific quantitative thresholds remain undefined, these vulnerable life stages warrant closer and more frequent clinical, nutritional, and biochemical monitoring. Preventive management should therefore adjust dietary calcium provision and UVB access according to physiological demand, with enhanced monitoring implemented in these high-risk groups [13,97].

Social structure within captive colonies may further influence disease risk. Dominance behaviors can restrict access to food and light, resulting in uneven nutrient intake and UVB exposure. Preventive management should therefore include multiple feeding and roosting sites, reduced overcrowding, and routine monitoring of hierarchical interactions to promote equitable resource distribution and minimize chronic social stress [13,97].

Environmental and activity-related factors contribute significantly to skeletal health. Limited flight space or inadequate environmental enrichment may result in reduced mechanical loading, favoring disuse osteopenia. Enclosures should include ample horizontal and vertical space for flight, along with hanging enrichment and variable roosting areas to encourage natural locomotion and muscular activity [13,97].

Another way to prevent the disease is through regular blood tests, which allow early detection of changes in bone health. Periodic evaluation of calcium, phosphorus, and alkaline phosphatase levels enables the identification of metabolic imbalances before obvious clinical signs appear [21]. Serum 25(OH)D_3_ can also be measured using relatively inexpensive RIA assays or, more recently, by LC-MS/MS, which also enables detailed profiling of the vitamin D metabolome, including 1,25(OH)_2_D_3_ and several catabolic metabolites [106]. Such metabolite profiling has proved helpful in the differential diagnosis of vitamin D-related hypercalcemia in humans and, although not yet routinely applied in veterinary medicine, could help distinguish nutritional vitamin D deficiency from primary disturbances of vitamin D metabolism in captive bats in the future.

The main potential risk factors, grouped by category, their effects, and corresponding preventive strategies are summarized in Table 1.

Although preventive management strategies are discussed at the genus level, most available evidence derives from observations in *P. vampyrus*, particularly juveniles. Consequently, dietary, environmental, and UVB-related recommendations should be applied with caution to other *Pteropus* species, taking into account interspecific differences in natural diet, foraging ecology, roosting behavior, and physiological adaptation.

## 3. Future Perspectives

Future research on metabolic bone disease (MBD) in captive *Pteropus* species should move beyond descriptive case reports toward coordinated, hypothesis-driven, multi-institutional research programs. Although recent surveys have provided valuable initial documentation of disease occurrence in zoological collections [9], the limited number of animals, institutions, and geographic regions studied constrains inference regarding prevalence, severity, and progression. Comparative analyses across institutions, climatic zones, enclosure designs, and husbandry practices are therefore essential to identify the nutritional, environmental, and managerial variables most strongly associated with disease expression. Establishing standardized epidemiological frameworks, including dietary audits, UVB exposure assessment, clinical screening, biochemical profiling, and imaging-based bone evaluation, should represent a central objective of future research.

A major research priority is to elucidate the physiological mechanisms regulating bone metabolism in *Pteropus* spp. and determine how they diverge from those described in other mammals and in hibernating microchiropterans. In hibernating bats, serotonin and calcitonin are co-stored within parafollicular cell granules, and their synthesis, granule maturation, and secretion exhibit marked seasonal fluctuations. These observations support the existence of a serotonergic–calcitonergic axis relevant to calcium homeostasis and bone remodeling [107,108]. Whether comparable dual storage or stress-related modulation occurs in non-hibernating megachiropterans remains unknown. So, investigating these pathways in *Pteropus* spp., particularly under conditions of mineral imbalance, low UVB exposure, or altered diet, may reveal endocrine regulatory mechanisms that have not yet been characterized in veterinary physiology.

Endocrine stress pathways also warrant focused investigation. Seasonal studies in free-ranging microbats demonstrate pronounced cyclic modulation of adrenal activity, with cortisol concentrations peaking during hibernation and remaining elevated relative to active periods. These findings challenge the assumption that energy-saving states are accompanied by endocrine suppression and instead point to dynamic seasonal resetting of the Hypothalamus–Pituitary–Adrenal (HPA) axis [109]. Although *Pteropus* species do not hibernate, they are exposed to chronic captivity-associated stressors (e.g., limited flight space, social restructuring, nutritional fluctuations) [110], which may similarly influence adrenal output. Evidence from microchiropteran models also highlights the marked skeletal plasticity of bats, including rapid osteoclast activation following hibernation and reproductive-associated fluctuations in cortical thickness, trabecular density, and bone turnover [111,112]. Whether comparable adaptive remodeling cycles occur in *Pteropus* spp. and whether captivity disrupts these processes remain unknown. Interactions among bone remodeling, reproductive status, lactation-associated mineral demands, stress hormone rhythms, and dietary composition, therefore, represent promising targets for longitudinal investigation.

Behavioral ecology represents another critical and underexplored dimension of MBD risk in captive colonies. In group-housed flying foxes, access to UVB radiation, nutrient-rich food, and optimal roosting sites may be unevenly distributed due to dominance hierarchies, creating chronic microenvironments of deprivation for subordinate individuals. Investigating how social structure, enclosure microclimates, spatial distribution of resources, and animal-to-animal interactions influence mineral homeostasis may inform the development of more equitable husbandry systems. Emerging technologies, including automated behavioral tracking and wearable UVB sensors, offer practical tools for quantifying these dynamics in real time.

Future studies should also adapt diagnostic concepts adapted from the CKD-MBD framework, particularly regarding the integration of biochemical markers, bone imaging, and tissue-level evaluation [25,26]. As recommended in earlier chiropteran work [52], the systematic implementation of radiographic and histopathological assessments in clinically affected individuals would significantly advance understanding of structural bone changes, disease staging, and treatment response. Although renal disease is not a recognized driver of MBD in flying foxes, adopting structured diagnostic approaches, such as a combined assessment of Ca, P, PTH, FGF23, vitamin D metabolites, and bone density, could help differentiate nutritional disease from rare cases involving renal or endocrine dysfunction. This mirrors KDIGO’s emphasis on multidimensional evaluation and may facilitate the development of species-specific clinical pathways for bats. However, a significant knowledge gap concerns the absence of species-specific reference intervals for calcium, phosphorus, 25(OH)D_3_, 1,25(OH)_2_D_3_, PTH, FGF23, alkaline phosphatase, and bone mineral density. Developing these baselines through large-scale sampling will enhance diagnostic accuracy and facilitate early detection of subclinical mineral imbalance. Incorporating advanced analytical tools, such as liquid chromatography–mass spectrometry for vitamin D metabolomics [71], CT-based bone microarchitecture assessment [24], and Dual-energy x-ray absorptiometry for mineral density quantification [76], would substantially refine clinical interpretation and inform preventive health programs.

A further priority for future research is the identification of physiologically meaningful exposure ranges and nutritional targets associated with skeletal health and disease risk. Such ranges are unlikely to be uniform across life stages, as juveniles undergoing rapid skeletal growth and females during pregnancy and lactation likely exhibit higher mineral and vitamin D requirements and increased sensitivity to UVB availability. Stratifying future datasets by developmental stage and reproductive status will therefore be essential to identify life-stage-specific risk windows and to develop targeted preventive strategies.

Inter-specific variation in natural diet composition, foraging ecology, roosting behavior, and sun exposure is also expected to influence mineral metabolism across the genus *Pteropus*. Regional differences in husbandry practices, enclosure design, seasonal light availability, and locally available food resources further contribute to inter-institutional and interspecific variability in disease expression. Future multi-institutional studies should therefore stratify data by species, geographic region, and climatic context to refine species-specific and locally adapted management recommendations.

Future research should also investigate genetic variability in vitamin D metabolism, calcium transport, and endocrine regulation, as well as the influence of developmental timing on disease susceptibility. Longitudinal studies beginning in early postnatal life, combined with field-based research integrating behavioral observation, environmental UVB measurements, and dietary analysis, are needed to determine whether transient deficiencies during critical growth windows predispose individuals to permanent skeletal pathology.

Collectively, these research directions have the potential to substantially refine understanding of metabolic bone disease in flying foxes, support evidence-based refinement of husbandry practices, reduce disease incidence, and improve welfare and reproductive success in ex situ *Pteropus* populations worldwide.

## 4. Conclusions

Metabolic bone disease in captive *Pteropus* spp. remains a significant welfare and conservation concern, most consistently associated with nutritional imbalance, insufficient ultraviolet B (UVB) exposure, and increased physiological demands during growth, reproduction, and captivity-associated stress. Given the predominantly observational and comparative nature of the available literature, the conclusions of this narrative review should be interpreted as associative and mechanistic rather than predictive or causative. Although experimental and longitudinal studies in *Pteropus* species remain scarce, the evidence reviewed here supports early recognition of mineral imbalance, correction of dietary calcium-to-phosphorus ratios, and provision of consistent, adequate UVB exposure as central components of preventive management to reduce irreversible skeletal damage.

A more systematic diagnostic approach, integrating biochemical profiling (including calcium, phosphorus, 25-hydroxyvitamin D_3_, and parathyroid hormone), imaging, and histopathology in advanced cases, would substantially improve clinical decision-making and enable earlier intervention. For zoological institutions, practical management actions include implementing standardized dietary audits, ensuring consistent access to UVB or appropriate vitamin D_3_ supplementation, closely monitoring juveniles and reproductive females, and minimizing social and environmental stressors that may exacerbate metabolic instability.

Integrating the mechanistic insights described in this review with epidemiological surveillance and applied husbandry research will enable evidence-based refinement of management protocols, reduce the incidence of MBD, and enhance welfare and reproductive outcomes across ex situ flying fox populations.

## Figures and Tables

**Table 1:** Preventive Measures for Metabolic Bone Disease in Captive Flying foxes (↑ increase; ↓ decrease).

Category	Risk Factors	Effect	Prevention
Nutrition	Low Ca Low protein Ca:P inverted (1:2) Fruit-only diets	Hypocalcemia ↑ PTH Poor bone mineralization	Balanced Ca:P ≈ 2:1, Pellets, pollen, leafy greens Ca supplements
UVB/Light	Indoor housing Glass panels Weak/old UVB lamps	↓ Vit D_3_ synthesis	Natural sun, if possible Reptile/bird UVB lamps 280–315 nm
Life stage	Growth Gestation Lactation	Higher mineral demand	Adjust diet Adjust UVB intensity Monitoring
Social structure	Dominance Competition for food/light	Uneven nutrient/light access Chronic stress	Multiple feeders/roosts reduce overcrowding
Environment & activity	Limited flight space, Poor enrichment	Disuse osteopenia Low bone strain	Provide a flight room Hanging enrichment Varied roosts
Medical	Renal/hepatic disease	Impaired Vit D metabolism	Routine blood analysis Early detection

## Data Availability

No new data were created.
